# Bat Cave Vulnerability to Anthropogenic Factors: Status and Priorities for Conservation Within the Mount Elgon Region, Uganda

**DOI:** 10.3390/life15121940

**Published:** 2025-12-18

**Authors:** Aggrey Siya, Benard Matovu, Lillian Nalukenge, Micheal Mutebi, Betty Nalikka, Kevin Castle, Tanya Dewey, Kalani M. Williams, Natalie R. Wickenkamp, Emma K. Harris, Innocent B. Rwego, Eric Sande, Charles Masembe, Rebekah C. Kading, Robert M. Kityo

**Affiliations:** 1 Department of Zoology, Entomology and Fisheries Sciences, Makerere University, Kampala P.O. Box 7062, Uganda; wamalamatovuben@gmail.com (B.M.); lilianpauline95@gmail.com (L.N.); micheljack146@gmail.com (M.M.); bnalikka@gmail.com (B.N.); ericsandephd@gmail.com (E.S.); cmasembe@gmail.com (C.M.); kityrob@gmail.com (R.M.K.); 2 Uganda Wildlife Research and Training College, Kasese P.O. Box 173, Uganda; 3 Wildlife Veterinary Consulting LLC, Fort Collins, Livermore, CO 80536, USA; castlekt@gmail.com; 4 Department of Biology, Colorado State University, Fort Collins, CO 80523, USA; tanya.dewey@colostate.edu; 5 Department of Microbiology, Immunology, and Pathology, Center for Vector-Borne Infectious Diseases, Colorado State University, Fort Collins, CO 80523, USA; kalani.williams@colostate.edu (K.M.W.); natalie.wickenkamp@colostate.edu (N.R.W.); emkate.harris@colostate.edu (E.K.H.); rebekah.kading@colostate.edu (R.C.K.); 6 Department of Biosecurity, Ecosystems and Veterinary Public Health, College of Veterinary Medicine Animal Resources and Biosecurity, Makerere University, Kampala P.O. Box 7062, Uganda; rwegovet@gmail.com

**Keywords:** chiropteran, karsts, subterranean, anthropogenic, human population, human–bat interaction

## Abstract

Uganda is home to a rich diversity of bats, which carry high ecological and socioeconomic value through the ecosystem services that they provide. However, critical bat habitats, including caves, are facing increasing anthropogenic pressures, and the types and frequencies of disturbances to cave-roosting bats are not well understood in Uganda. Therefore, we examined the role of anthropogenic disturbances in caves to assess the threats posed to bat populations. We used the Bat Cave Vulnerability Index (BCVI) framework to score 14 caves inhabited by bats within the study region. We included qualitative surveys with human communities to better understand various aspects within the BCVI. All bat species recorded were of the IUCN category “Least Concern”. The BCVI indicated 50% of the caves (with insectivorous and frugivorous bats) require urgent conservation interventions due to high bat diversity and anthropogenic disturbances (e.g., guano collection). Most of the caves studied were highly vulnerable to anthropogenic disturbances, as assessed in the study. All the caves we studied (except two) are outside the protected area, and due to their imputed vulnerabilities, interventions ought to be implemented to balance cave conservation and human use in the Mt. Elgon area. Such interventions should integrate human factors.

## 1. Introduction

Bats play critical roles in sustaining ecosystems and human livelihoods. This is enabled by their high diversity, i.e., over 1500 species worldwide, with over a fifth of them in Africa [1,2]. In terms of ecological roles, they feed on insects [3,4], fruits [5,6], nectar [7], blood and vertebrate animals [8,9], and generate nutrient-rich guano [10,11]. These services are critical to sustaining human livelihoods as they support different livelihood assets, i.e., spiritual and cultural relations and food production, among others [12,13]. To exemplify these values, in North America, bat pest predation services have been predicted at USD 3.7 billion/year in agricultural fields [14]. Meanwhile, in Asian countries, e.g., Indonesia, bats have been indicated to reduce pests in cacao plantations, sustaining productivity [15]. On the same note, reports from several African countries indicated critical socioeconomic values of these mammals. For example, the South African macadamia farms benefit from bats through pest predation [16]. Meanwhile, in Uganda, bats are an important source of guano, which is utilized in agricultural fields to support crop production [17]. This implies their critical role in ensuring food security across different scales. Besides the food security dimension, in Uganda, bats are used in a range of sociocultural practices, e.g., considered as a totem in some clans [13,18]. These are crucial aspects that bring humans closer to bats and have implications for the health of both.

Despite the different values, bat populations are threatened by anthropogenic factors, including pollutants and habitat change [19,20,21]. Cave ecosystems that are critical roosting sites for bat populations are commonly severely affected by anthropogenic activities [22,23,24]. Anthropogenic cave disruption includes noise, light, and actual cave destruction [25,26]. These threats may undermine the population of bats across different scales. The International Union for Conservation of Nature estimates that over 15% of species are considered endangered or near threatened (IUCN), with their populations decreasing globally [27,28,29]. This is especially common in tropical regions where anthropogenic activities cause land use changes, threatening bat populations [30,31]. Moreover, tropical regions host substantial bat diversity, contributing to the sustainability of ecosystems [25,32]. Therefore, there is a need for interventions to mitigate threats to bat populations in tropical regions and elsewhere. These partly require understanding bat vulnerability within cave ecosystems. Vulnerability studies have been undertaken in Bulgaria [24], Philippines [26] and Ghana [33] providing critical information that can be utilized to support conservation of bat populations. Beyond bat populations, such information provides opportunities for understanding the role of humans in landscape changes. Notably, bats are diverse and provide a model for a better understanding of alterations within ecosystems across different scales [19,34,35].

Caves are critical in sustaining bat populations as they are used for roosting [36], breeding [37], hibernation, and swarming [38]. Bats choose and use different caves, depending on the environmental and geographical features [39]. Notably, temperature and humidity can influence the selection of caves for breeding and/or hibernation (especially in temperate regions) among other functions [37,40,41]. Cave selection by bats varies across space and time depending on the species of the bat and foraging behavior among other factors [42,43]. While natural factors influence ambient conditions in caves, human activities can significantly make them unfit for bat utilization [44,45,46]. The vulnerability of caves is critical as bats are also good candidates as surrogate species (i.e., keystone, umbrella, and indicator taxa) of cave biodiversity and conservation value [47,48]. Adequate scientific data that provides an understanding of the vulnerability of cave bat roosts to anthropogenic disturbances provides opportunities for designing suitable conservation interventions that try to accommodate human access needs while protecting the most vulnerable aspects of the cave ecosystem for bats. This approach contributes to sustaining ecosystems and human livelihoods. Accordingly, we assessed the vulnerability of caves used for roosting by bats to anthropogenic disturbances within Mount Elgon, Uganda. This study was guided by two questions, i.e., (i) how do Biotic Potential and Biotic Vulnerability vary for caves inhabited by bats within the Mount Elgon region of Uganda? and (ii) what caves should be prioritized for conservation within the Mount Elgon region of Uganda? These questions included an assessment from a human dimensions perspective as described in the methods.

## 2. Materials and Methods

### 2.1. Study Area

**Figure 1:**
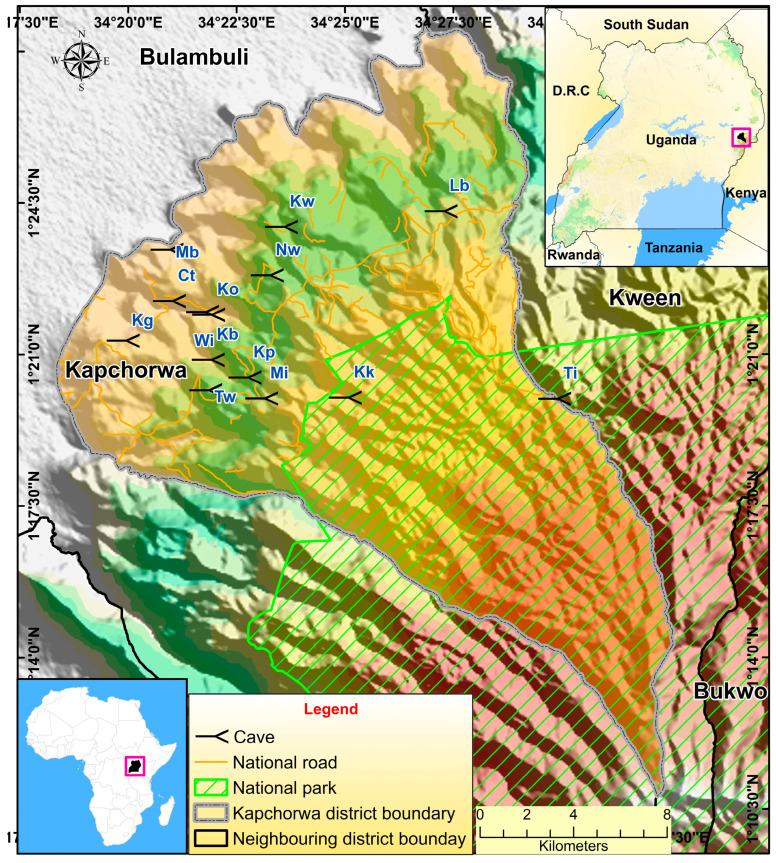
Map of the study site. Specific caves studied are denoted by two-letter abbreviations (instead of full names) in order to protect cave identity for conservation purposes.

This study was undertaken on bat-occupied caves in the Mount Elgon region of Uganda, with a focus on the Kapchorwa district, with location coordinates of 1.35° N, 34.38° E (Figure 1). Kapchorwa district is bordered by Kween District to the northeast and east, Sironko District to the south, and Bulambuli District to the west and northwest (Figure 1). Kapchorwa has the largest human population (133,621) within the region, with a rapid growth rate of 2.5% (almost equivalent to the national rate 2.9%) [49]. The district also has very diverse economic activities, including tourism, agriculture, and trade, among others [50].

### 2.2. Study Design and Approaches

A cross-sectional survey design was applied in communities near cave sites, utilizing mixed methods research approaches [51,52]. Mixed methods allowed for data integration, thus providing a better understanding of the research question [53,54]. The purpose of the community engagement was to gather insights into the activities undertaken in the caves. This insight provided by the communities would then validate the findings of the vulnerability factors identified for the caves. Vulnerability factors considered included those that are observable and can be recorded. These factors also included those that the community members (near each cave) would report. Both quantitative and qualitative data were collected.

### 2.3. Study Population

This study focused on both insect and fruit-eating bats inhabiting caves whose populations formed the basis of the quantitative data (see Table 1).

The measured parameters within each cave included bat numbers, bat species richness, and human activities within caves. Households neighboring caves inhabited by bats formed the target population for qualitative data collection through focus group discussions (FGDs). FGDs focused on community perspectives about activities carried out within each of the caves. Two focus group discussions were organized in villages near each cave involving persons who live nearest to the caves. The distance to each cave from members of the local community was variable, and we considered those that were closer until we reached a maximum of 10 individuals. The FGDs were held with adult (at least 18 years old) male and female participants after verbal consent was obtained. The consenting process included discussion of information shared about the study, participation, and privacy practices. Participants were also informed of the study’s purpose, procedures, potential risks, benefits, and their right to withdraw at any time.

### 2.4. Sampling Strategy and Data Collection

Only caves that were easily accessible (by the research team) within the district were selected for this study. Bat counts in the selected caves were performed over a period of 3 years (2022–2024) whenever the caves were visited for routine monitoring studies on the bats as part of other projects. At each cave, bat species in the caves were identified, and counts of individuals were conducted using either visual counts or mist-netting to estimate population sizes. As some caves had several chambers, counting every single individual was difficult, in which case an estimated maximum number was recorded. Using these approaches, all the parameters were collected to describe Biotic Potential (BP) and Biotic Vulnerability (BV), as described by Tanalgo and colleagues [87].

In addition to bat-specific data, anthropogenic source data, including cave accessibility, cave size and openings, the effort of exploration, tourism potential, cave use, land-use activities in areas adjacent to the cave, and the presence of temples and sacred structures in the caves were also investigated. Literature was used to gather data on some Biotic Potential (BP) parameters that included species endemism (E) and conservation status (cons).

For prioritization of caves and suitable conservation interventions to sustain populations of bats, data were collected on the potential threats to the different bat roosts. This was undertaken using a checklist that covered anthropogenic and natural factors that could threaten bat populations (Table S3 in Supplementary File). For instance, the checklist covered the presence of collapsed entrances, household items, and introduced twigs, among others.

### 2.5. Data Analysis

The Bat Cave Vulnerability Index (BCVI) was used to assess the vulnerabilities of these caves to anthropogenic disturbance [87]. The BCVI is described by the Equation

BCVI=(BV) × (BP)

where BCVI = Bat Cave Vulnerability Index, BP = Biotic Potential Index, and BV = Biotic Vulnerability Index.

### 2.6. Computation of Biotic Vulnerability (BV)

Biotic Vulnerability (BV) represents cave geophysical features and anthropogenic threats to the cave.

The Biotic Vulnerability (BV) value is derived using the Equation

$$BV=ƩN/N^{0}$$

where N = threats assessed, and N^0^ = number of threats assessed/present,

Threats to each of the caves were obtained through observations and interviews with community members. These respective threats were scored using a scale of 1–4 (as outlined in Table S3 in the Supplementary). Threats were then averaged to obtain the value of Biotic Vulnerability (BV). The value computed using the Biotic Vulnerability (BV) index was assigned to a category of “A”, “B”, “C”, or “D”, following the methods of Tanalgo et al. [87]. The lowest value of this index is 1.00 as ‘Status A’, which represents caves that are highly disturbed and/or prone to disturbance. The highest value is 4.00 as ‘Status D’, which represents pristine caves with no disturbance (Table 2, as in [87]).

### 2.7. Computation of Biotic Potential (BP)

The following metrics were integrated: Biotic Potential (BP) of the caves, species richness (S) and Population (A), species relative abundance (Ar), endemism (E), and conservation status (cons), and species-site commonness index (site) (see [87]). These variables are included in the Equation

BP=Ʃ Species 1 [(A×Ar×E×cons×site)+Species 2 (A×Ar×E×cons×site) … Species nth (A×Ar×E×cons×site)] (S)

In this study, the method for assessing bat populations was standardized among all cave sites. This included using the maximum number counted of each species from all the counts conducted or estimated over the three-year period, i.e., 2022 to 2024, in the BP calculations (Table S1 in Supplementary). The species richness (S) represents the number of bat species encountered in the caves.

The relative species abundance (Ar) indicates information on the status of the population relative to other caves. This value was calculated for each cave by dividing the maximum number of bats observed in that cave by the sum of the maximum numbers of bats of that species observed from all caves. By exploring relative abundance for each species, it provided a better understanding of how each cave performed relative to “global/regional” species average, and not the caves of particular importance [87]. However, because accurate counts of bats in the cave roosts were difficult to establish, the population figures presented may be under-representing the population of bats in some of the caves.

The endemism value (*E*) is based on bat species range distribution, while conservation status (*cons*) is based on the global population, distribution status, as well as trends of bat species as described by Tanalgo and colleagues (see [87]). These two variables were derived from the International Union for Conservation of Nature (IUCN), where respective factors were given scores. For this paper, we adopted the scale utilized by Tanalgo and colleagues to indicate the scores for the endemism and conservation status of different bat species (see [87]). The Species-site commonness index (*site*) is a measure of the rarity of the bat species from the caves assessed and is calculated as the frequency of occurrence of each bat species in the caves assessed [87]. A species-site commonness index of less than 1 is interpreted as the species only occurring in very few caves, whereas values equal to 1 reflect the presence of that species in all caves. For this study, the Biotic Potential (BP) values were set ranging from 100 and below to 900 and above. Consequently, “Level 1” caves were classified as high in species diversity, while “Level 4” caves were classified as the least biodiverse caves (Table 3).

The results from both indices, Biotic Potential (BP) and Biotic Vulnerability (BV), were multiplied to form an alphanumeric value that summarizes the general vulnerability and priority of the cave. When both indices are synergistically joint (alphanumeric index), different prioritization levels are derived.

Shannon–Weiner index of diversity was computed for each cave using the Equation

H=−∑pi ln pi

where pi is the proportion of each bat species, ln pi is the natural logarithm of the value of the proportion of each species in a sample. H is the diversity index.

### 2.8. Determining Bat Cave Vulnerability Indices (BCVI)

Cave sites were classified based on the combined values of BP and BV using criteria described by Tanalgo et al. [44]. Caves rated as ‘1A, 1B, and 2A’ (High Priority) are especially vulnerable to anthropogenic threats and population declines and represent locations where conservation interventions should be implemented to control human activities. Additionally, caves rated as ‘1C’, ‘1D’, ‘2B’, ‘2C’, ‘2D’, ‘3A’,’3B’, ‘3C’, and ‘3D’ are categorized as moderately vulnerable due to less frequent anthropogenic pressures and disturbance. Lastly, caves that are ranked as ‘4A’, ‘4B’, ‘4C’, and ‘4D’ are considered the least vulnerable (Low Priority) because they either have high populations of bats and/or are relatively undisturbed. All calculations were performed in Microsoft Excel 2023 for Windows, Redmond, Washington, USA [88]. Chi-square and Kruskal–Wallis rank sum tests were computed for the different bat species at 95% confidence level.

Statistical tests were conducted using two complementary approaches, i.e., rank-based (non-parametric analog) and count regression (model-based). Because the same caves were observed across multiple species, we assessed whether counts differed by species within caves using a rank-based approach. Counts were ranked within each cave, and an ANOVA model of ranks was fitted with species and cave fixed effects (equivalent in spirit to a Friedman-style blocked comparison). To jointly test differences across species and differences across caves while respecting the count nature of the outcome, we estimated the Poisson regression with species and cave fixed effects and robust standard errors. Given clear over-dispersion (Poisson goodness-of-fit rejected), we also estimated a negative binomial model with the same fixed effects and robust standard errors. A Joint Wald test was conducted to determine the differences in all species indicators at each cave and also among the species. Species-specific cave variation was determined using a Kruskal–Wallis test because cave-fixed-effects Poisson models may fail to converge for extremely sparse species (e.g., one non-zero cave and zeros elsewhere).

Qualitative data from FGD, which were collected during the study, were analyzed using thematic approaches. This included the 6-step process, i.e., familiarization, coding, theme development, reviewing themes, defining themes, and reporting findings. Familiarization included understanding the data the way it is. At the coding stage, coding was assigned to the different key ideas, patterns, and concepts. Thereafter, codes were grouped together into a theme reflecting broader patterns of data. These themes were grouped together to form broader themes. The developed themes were then reviewed and updated over the analysis period to effectively capture the different aspects of the data. After reviewing and refining themes, their consistency was ensured by providing clear definitions and names. Finally, the findings were reported with quotes and an interpretation of the meaning of such quotes. This six-stage process was carried out with the support of NVivo version 12, USA, Massachusetts [89].

## 3. Results

### 3.1. Overview of the Caves Assessed

In total, fourteen caves were assessed within the study area (Table 4 and Table S1 in Supplementary). The total number of bat species in the caves was seven, including five insectivorous, i.e., *Coleura afra* (Peters, 1852), *Miniopterus* spp., *Rhinolophus* spp., *Hipposideros caffer* (Sundevall, 1846), *Nycteris macrotis* (Dobson, 1876), and two fruit-eating bats, i.e., *Rousettus aegyptiacus* (E. Geoffroy, 1810), and *Myonycteris angolensis* (Bocage, 1898) species (Table S1 in Supplementary File). For this study, *Rhinolophus* spp. and *Miniopterus* spp. bats are taken as one species owing to the complexity of characteristics of these genera, and the difficulty in separating species using the observational methods used in this study. In terms of conservation status, all the bats recorded were in the “Least Concern” IUCN category (Table S3 in Supplementary File). The *Rhinolophus* genus was included in the IUCN “Least Concern” category because all the potential species present in our study area fall within this category. All caves experienced at least one of the human activities (disturbances), including mining for guano and rocks, tourism, sociocultural activities, and bat hunting (Table S2 in Supplementary File).

Bat species varied significantly across the caves based on both rank-based analysis (F(6,78) = 3.01, *p* = 0.0106) and in count regression models (Poisson FE χ^2^(6) = 44.67, *p* < 0.001; NB FE χ^2^(6) = 19.50, *p* = 0.0034) (Table S5 in Supplementary Material). Similarly, cave differences were significant in both Poisson and negative binomial fixed-effects models (Poisson FE χ^2^(13) = 179.33, *p* < 0.001; NB FE χ^2^(13) = 63.23, *p* < 0.001) (Table S6 in Supplementary Material), but significant within-species differences were not detected in this small dataset. Species-specific Kruskal–Wallis tests did not show significant cave variation for any individual species (all *p* = 0.4478 with ties), likely reflecting sparse counts and many tied values (Table S7 in Supplementary Material).

### 3.2. Biotic Vulnerability, Biotic Potential, and Level of Conservation Priorities for the Different Caves Inhabited by Bats

In terms of biotic potential, four of the caves ranked “Level 1”, reflecting the high number of bats (Table 5). The other caves had Biotic Potential of “Levels 3 and 4” (Table 5). In terms of biotic vulnerability, over half (71.4%, n = 14) of the caves were in category “A” of the biotic vulnerability, reflecting greater accessibility and human activities carried out within these caves (Table 5). The combined BCVI revealed that up to half (50%) of the caves assessed ranked “High” or “Moderate” priority for conservation (Table 5). These rankings suggest that these caves are critical in supporting bat populations and therefore require high prioritization for conservation action.

### 3.3. Human Dimensions

**Figure 2:**
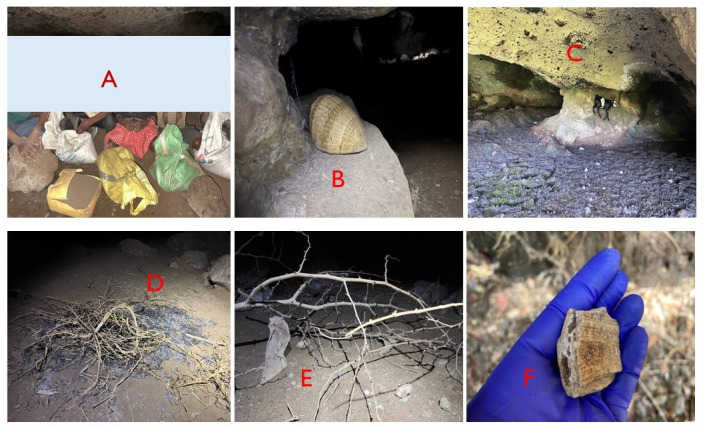
Photos depicting some of the anthropogenic activities within the different caves, i.e., guano mining (**A**), cultural practices (**B**), shelter for domesticated livestock (**C**), social activities (**D**), bat trapping/hunting (**E**), and mining of rock crystals (**F**).

From the community engagements, it was clear that caves are widely utilized by communities for various activities. This mainly included their use for shelter, sociocultural and spiritual activities, as well as salt and guano mining (Figure 2).

These uses present opportunities for increased vulnerability of bats to human disturbances. Notably, guano mining appeared to be commonly practiced, increasing human presence in caves. This increased human presence in caves potentially causes disturbances to bat populations. Additionally, cultural and spiritual activities were undertaken in caves. Other activities included keeping livestock and hunting for bats. All of these activities pose a disturbance to bat populations inside caves.

Within the caves, which were ranked as high and moderate priority, some of the human activities that were observed and also reported during focus group discussions included the following:

- Guano mining was mainly in Kw, Ti, Nw, and Kp. The mined guano is used in banana and coffee plantations as fertilizer for the crops.

*“We get a lot of ‘buresik’ from this cave. You can get a full sack of 100 kgs and can enable production of big bunches of bananas”*, one male elder noted regarding “Nw” cave.

- Some sociocultural activities that were recorded in the caves included childbirth, as well as initiation. These were indicated by the objects found in the caves, as well as interviews with local residents. This was mainly reported in Nw and Kw.

*“Women who give birth to twins are brought here with some food produce carried using ‘Kiiset’ (locally made container using bamboo). The women are the ones who do this to their fellow women, and they sing while the drum is beaten. This is done to protect the children and enable them to live longer”*, one female elder noted regarding “Kw” cave.

- Most caves are utilized as shelter whenever it rains. This was reported to be a common practice for those who cultivate the land near the caves, as well as those engaged in livestock rearing. This use was reported to happen more frequently in the wet season. This was reported in all the caves.

*“For us, whenever it rains, we go to the cave because that’s the nearby house. Rain cannot, of course, get you when you are inside”*, noted by one male elder regarding “Kp” cave.

*“Whenever we are in the lower altitude, we construct an animal shelter near the cave, and we sleep inside. This is good because the cave is protective from hostile enemies”*, one male respondent noted regarding “Nw” cave.

### 3.4. Conservation Priorities

**Figure 3:**
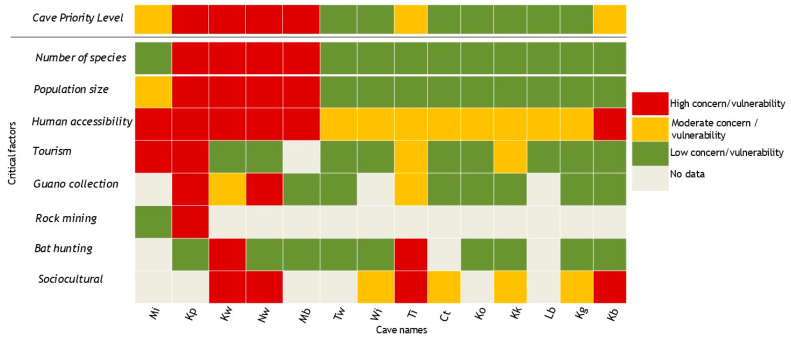
Priority ranking of caves assessed (critical factors are threats to the cave, with colors depicting a scale from 1 to 4, with 1–white; 2–green; 3–yellow; 4–red). Priority levels for the caves indicate the level of urgency for conservation. Conservation priorities rank from 1 to 4 (with 1–white; 2–green; 3–yellow; 4–red), with 1 being the least priority and red being the top priority.

Our results show that half of the caves in the study site (7/14 or 50%) were in the “High” and “Moderate” conservation priority categories (Figure 3). The main critical factors linked to these high and moderate conservation priorities included the high number of bat species and high human presence inside and outside the cave. Our results identified four caves (Kp, Kw, Nw, and Mb) to be of high conservation priority, a fact we attribute to more species in the cave and human presence. Focus Group Discussions with members of the local community near some of the caves (i.e., Nw, Mi, Wi, and Kw) suggested they were historically used as shelter for livestock (mainly cattle). However, this activity is no longer happening.

*“We used to use this cave for keeping our animals and protecting them from raiders, but now there are many crops around, so we have moved our cows further down the lower belts”*, one male elder noted regarding “Nw” cave.

*“This cave would accommodate cows for the whole community. It is so big, that is why you see the cow dung is still a lot”*, another male elder added regarding “Nw” cave.

## 4. Discussion

The results of this study revealed that a high number of caves of the Kapchorwa area of Mount Elgon, Uganda, are exposed to anthropogenic disturbances, determined in terms of biotic vulnerability. All the caves that were highly vulnerable to anthropogenic factors had both insectivorous and frugivorous bats.

Critical anthropogenic drivers for cave vulnerability included the use of caves for tourism, accessing guano, and undertaking cultural and spiritual events. In some cases, there were incidences of bat hunting activities evidenced by thorny twigs used for bat capture and information from community surveys. Evidence of fireplaces was recorded in the caves, and community members indicated that some bats hunted are prepared onsite before being taken out to the community. Indeed, most of the caves that ranked high priority for conservation experienced hunting. These are critical anthropogenic threats to cave biota and have the potential to affect bat populations. Declines in bat populations would threaten ecosystems within such sites, as bats are indicated to be umbrella species [47,48]. Notably, the nutrient-rich guano provides energy to microbes that, in turn, support other organisms within the cave [47,48].

A study in Costa Rica found human activities, including tourism, threaten cave systems [90]. A study in the Philippines found hunting, tourism, and guano mining to be critical factors affecting cave-dwelling bats [26]. The frequent human visits have also been shown to increase noise disturbances, which cause bats to fly, losing energy while disrupting echolocation [91,92]. Such noise has been shown to affect the foraging and roosting behavior of insectivorous bats [93]. Some studies shown that noise alters the gene expression associated with diseases and metabolism in bats [94]. Therefore, while our study did not attempt to analyze the effects of human disturbances on bats, it is possible that such occurrences pose wide-ranging effects on bats within cave ecosystems. Interventions thus ought to be implemented to mitigate such incidences to protect bat populations across different scales. This will contribute to sustaining ecological systems in and outside of caves.

The bat fauna in the caves studied included both insectivorous and frugivorous species, largely occurring in different associations in the different caves. Species recorded in Kapchorwa caves have been previously reported within this region, as well as other areas [95,96]. It is therefore critical to manage these caves already occupied by bats to ensure the survival of the different species and their populations across their range [97,98,99]. This is fundamental in protecting this mammalian order, as it is known to face serious threats. For instance, according to the International Union for Conservation of Nature (IUCN), 25, 87, and 110 bat species are listed as “Critically Endangered” (face imminent risk of extinction), “Endangered”, and “Vulnerable”, respectively [100]. Similarly, 15%, 18%, and 57% of the species globally are listed as threatened, data deficient, and with over half having unknown population trends [28]. These trends and data deficiencies reflect threats to these mammals, potentially undermining the flow of ecosystem services that are often associated with them [23,101]. The threats to bat populations are closely linked to the ways that human communities pursue their livelihoods [28]. For instance, hunting of bats (for consumption, medicinal, and souvenir purposes, among others) as well as agricultural activities have been noted to significantly undermine bat populations [28,102,103].

From the community engagement through FGDs, it was apparent that caves are central to socioeconomic aspects [13]. This socioeconomic connection to bats reflects their value in human societies, as observed elsewhere [104,105,106]. Such benefits derived from cave ecosystems, if not balanced, can have negative consequences on biota (e.g., bats) in such sites. For instance, human activities within cave and karst ecosystems have been indicated to be the major drivers of extinction for diverse cave-dependent species [46,107]. For instance, *Miniopterus* sp. and *Rhinolophus* sp. were documented to be extirpated as a result of anthropogenic factors within caves in Minami-Daito Island in Japan [108]. Similarly, in Puerto Rico, *Mormoops megalophylla* was extirpated as a result of microclimatic alterations within the caves, with anthropogenic factors being the main driver [109]. While our study did not attempt to analyze the extinction potential of the different bat species, the increased human activities in such sites present opportunities for loss of some species of bats. Moreover, some of the bats in our study site, including *Miniopterus* spp., fall in the same group as some of these species that are now extinct due to anthropogenic disturbances. This reflects the need to protect caves inhabited by such species. Therefore, to sustain bat populations in this region, interventions that integrate human dimensions and promote conservation messaging that educate communities about the benefits bats provide can foster the sustainable management of caves in the Mount Elgon region. This approach will contribute to the sustainability of bat populations as well as other biota within caves.

In terms of priorities for conservation, 50% of the caves ranked “high” or “moderate” priority for bat conservation, reflecting the incidences of broader socioeconomic activities on the cave ecosystems currently supporting high bat populations within this area. While similar studies are yet to be undertaken in Uganda and utilizing similar methodologies, the results obtained in this study are comparable to those in other areas. For instance, in Ghana, a study by Nkrumah and colleagues indicated caves to fall mainly within “high” and “moderate” conservation priorities [33]. Similarly, a study in Bulgaria by Deleva and colleagues revealed 32% of the bat roosts fall within the high-priority conservation status [24]. In Costa Rica and the Philippines, some caves fell into the “high priority” conservation category [38,64]. While the scales for analyzing different parameters within the framework were different, the results from previous studies, and ours, suggest that increasing pressures from anthropogenic drivers may negatively affect bat populations in cave ecosystems. This difference in scaling for the framework parameters was necessary as the nature of caves and socioeconomic and environmental factors are highly context-dependent. This context dependency implies that conservation interventions have to be adaptable to the local contexts for significant outcomes to be realized.

## 5. Strengths and Limitations of This Study

While the BCVI provides opportunities for adapting in different contexts through recalibration, it may be difficult to apply in areas where bats are migratory in nature. Such migrations of bats affect the parameters of the model, resulting in inaccurate priority indices. Integration of the human dimensions through focus group discussions enhanced our understanding of anthropogenic factors within these caves. During the implementation of conservation interventions, such human dimensions are useful in ensuring community participation, as well as the sustainability of the actions. Indeed, our study conducted in the same area indicated that communities were willing to support bat conservation through labor and monetary values [18]. Moreover, they can do this for the different ecosystem values derived from these mammals. This framework also provides an opportunity for extending conservation actions outside protected areas, given that caves already afforded some protection within the park had lower conservation priorities compared to those outside the park boundary. Moreover, some of the caves outside the park had more bat species and individuals compared to those inside the park.

Furthermore, considering only the maximum number of bats counted on different occasions may likely have underestimated/over-estimated the real populations in some of the caves. However, because of our repeated visits to the caves, the errors associated with this can be minimal, so it can have less effect on the final indices of the BCVI.

## 6. Conclusions and Recommendations

Our study highlights the vulnerability of a set of caves within Mount Elgon to anthropogenic disturbances, on the basis of which we highlight the need for targeted conservation actions to protect these unique habitats and their bat populations. Despite the absence of long-term monitoring data, current findings underscore the necessity of immediate interventions to sustain bat populations and maintain the ecological integrity of cave systems in the region. Working with members of the local community, it may be essential to implement urgent conservation interventions focused on the most vulnerable caves to safeguard resident bat populations. This can include interventions that limit human presence in the caves, e.g., fencing cave entry points and designating areas for viewing bats. As bats also play critical roles in human welfare, it is essential to design human livelihood strategies that align with the sustainability of cave ecosystems, ensuring that development does not compromise ecological balance. To reduce pressures on the caves and bat roosts, the community initiatives could consider opportunities to compensate for goods and services derived from bats and cave ecosystems. To better understand the integrity of caves and the stability of roosts and bat communities therein, given the continued community interests, long-term monitoring efforts will be essential to generate robust data to inform any conservation interventions. This will support understanding of the differentiated effects of anthropogenic factors on the bat species.

## Figures and Tables

**Table 1:** Characteristics of the different bats studied.

Bat Species Name	Distribution (Based on IUCN)	Population Status (Based on IUCN)	IUCN Red List Status	Feeding Habit	Roost Site Selection	Site Fidelity	Associated Disease-Causing Pathogens	Value to Human Communities and Ecosystems	Identified Threats to Its Population
*Coleura afra*	Regional Endemic (RE) (Africa–central, west, and eastern)	Unknown	Least Concern	Insectivorous [55]	Caves with stable climates and near water and forage resources [55,56,57]	Show high site fidelity [58]	Paramyxoviruses, e.g., *Belinga bat virus* [57]	Pest suppression [55,59]	Degradation of roosting sites, including land use and land cover change [55]
*Miniopterus* spp.	Regional Endemic (RE) (southern and eastern Africa)	Decreasing	-	Insectivorous [60]	Caves, karsts, and mines with stable climates and near water and forage resources [61,62,63]	Some (e.g., *Miniopterus schreibersii*) change roost sites [60], but others do not.	Paramyxoviruses [64] and Coronaviruses [65]	Pest suppression [60,66]	Human disturbances within caves and other roosting sites [67]
*Rhinolophus* spp.	Regional Endemic (RE) (Eastern Africa, tropical region, southern Africa, parts of northern Africa)	Decreasing	-	Insectivorous	Caves, karsts, and mines are inhabited by other species, e.g., *Miniopterus* spp. [64,68]. Uses human settlements as well [69]	Exhibits roost philopatry [70]	Coronaviruses [71,72]	Pest suppression [73]	Human disturbances within caves and other roosting sites [74]
*Rousettus aegyptiacus*	Widespread (NE) Africa and Asia	Stable	Least Concern	Frugivorous	Caves or artificial structures (e.g., abandoned buildings, tombs, and mines) [75]	Show site fidelity [76]	*Marburg Virus* [77]	Consume fruits supporting seed dispersal [78]	Human disturbances within caves [67]
*Hipposideros caffer*	Restricted to the southeastern part of Africa	Decreasing	Least Concern	Insectivorous	Caves, hollow trees, abandoned mines, and buildings [79]	Similar species show site fidelity [80]	Coronaviruses [81]	Consume pests [82]	Human disturbances within roosting sites [83]
*Myonycteris angolensis*	Restricted to the tropical region	Decreasing	Least Concern	Frugivorous	Caves and other hollow sites [84]	Like other megabats, they show site fidelity [76]	Rhabdoviruses [85]	Consume fruits (like other bats) supporting seed dispersal [78]	Human disturbances within roosting sites, e.g., caves [67]
*Nycteris macrotis*	Restricted to the tropical region	Unknown	Least Concern	Insectivorous	Caves, hollow trees, abandoned mines, and buildings [56]	Return to the site [86]	Coronaviruses [81]	Consume pests like others [82]	Human disturbances within roosting sites, like other insectivorous bats [83]

**Table 2:** Scale index for Biotic Vulnerability (BV) scored as per Table S2 in Supplementary Materials.

BV Score	Status	Probable Condition
1–1.99	A	Greater accessibility and are highly prone to human disturbance and activities
2–2.99	B	Lesser accessibility, but disturbance is/may be present in distance
3–3.99	C	Less accessibility, less prone to human disturbance
4.00	D	No disturbance, far from localities, and difficult to pass through

**Table 3:** Priority level for bat caves based on biotic potential.

BP Score	BP Status	Probable Scenario
900 and above	Level 1	Bat cave/s hold the highest numbers of species, relatively with the largest populations, with many threatened and endemic species, and with rarest species also represented.
500 to 900	Level 2	Bat cave/s are likely to have high species richness (>1 number of species), with large populations, and may contain a number of threatened and endemics, with some rare species.
100 to 499	Level 3	Bat cave/s are likely to have few species, relatively low populations, with few or no threatened and endemic species present. Most species present are common
100 and below	Level 4	Bat cave/s have a few species at very low populations, and most species are of least concern, non-endemic species, and are common in all cave sites

**Table 4:** Species recorded and their proportions in different caves.

Cave ID	Species Recorded in Each Cave and Their Relative Abundances							Number of Bat Species per Cave
	*Coleura afra*	*Miniopterus* spp.	*Rhinolophus* spp.	*Rousettus aegyptiacus*	*Hipposideros caffer*	*Myonycteris angolensis*	*Nycteris macrotis*	
Mi	0	0	0.0019	0	0	0.4955	0.0051	+++
Kp	0	0	0.1647	0	0.4261	0	0.2816	+++
Kw	0	0.1553	0.3107	0.1896	0.2273	0	0.1367	+++++
Nw	0	0.4678	0.3276	0.7257	0.2159	0	0.2765	+++++
Wi	0	0.0007	0.0650	0	0	0	0	++
Ti	0	0	0.0096	0.0847	0.0606	0.2162	0	++++
Ct	0	0	0.0183	0	0.0492	0	0.0235	+++
Tw	0	0.0084	0	0	0	0	0.0061	++
Kk	0	0	0	0	0.0208	0.2883	0	++
Ko	0	0	0.0226	0	0	0	0	+
Lb	0	0	0.0275	0	0	0	0.0673	++
Kg	0	0	0.0520	0	0	0	0.0592	++
Kb	0	0	0	0	0	0	0.1439	+
Mb	1	0.3678	0	0	0	0	0	++

+ denotes a single species of bats in each cave.

**Table 5:** Bat cave vulnerability indices for the different caves.

Cave ID	Shannon Diversity Index (*H*) for Each Cave	Biotic Vulnerability (BV) Value	Score	Biotic Potential (BP) Value	Biotic Potential (BP) Score	BCVI	Priority Setting (Interpretation)
Mi	0.362	1.0000	A	176	3	3A	Moderate
Kp	1.084	1.2857	A	3456	1	1A	High
Kw	1.411	1.2857	A	7035	1	1A	High
Nw	1.240	1.2857	A	24,319	1	1A	High
Wi	0.124	1.2857	A	75	4	4A	Low
Ti	0.820	1.2857	A	150	3	3A	Moderate
Ct	0.723	3.2857	C	36	4	4C	Low
Tw	0.441	1.2857	A	2	4	4A	Low
Kk	0.342	1.2857	A	42	4	4A	Low
Ko	0.058	1.2857	A	5	4	4A	Low
Lb	0.698	1.7143	A	71	4	4A	Low
Kg	0.653	2.1429	B	92	4	4B	Low
Kb	0.021	2.1429	B	130	3	3B	Moderate
Mb	0.685	2.1429	B	3617	1	1B	High

## Data Availability

Since the data with cave names can expose bats to increased human intensity, we will make it available on request from the corresponding author. Similarly, data from focus group discussions will be available on request.

## Supplementary Materials

The following supporting information can be downloaded at: https://www.mdpi.com/article/10.3390/life15121940/s1. Table S1: Caves and bat species recorded. Table S2: Biotic vulnerability of the caves. Table S3: Landscape and human-induced activities that influence vulnerability of caves. Table S4: Conservation status of the bats. Table S5: Tests for differences in bat counts across species (within caves). Table S6: Tests for differences in bat counts across caves (overall). Table S7: Species-specific variation across caves (Kruskal–Wallis tests).

## Abbreviations

The following abbreviations are used in this manuscript:

BCVI	Bat Cave Vulnerability Index
BP	Biotic Potential
BV	Biotic Vulnerability
FGD	Focus group discussion
