# Supplementary material for: Bat Cave Vulnerability to Anthropogenic Factors: Status and Priorities for Conservation Within the Mount Elgon Region, Uganda
Source: Life (Basel). 2025 Dec 18;15(12):1940. doi: 10.3390/life15121940 (PMC12735193; doi:10.3390/life15121940)
Supplement: Supplementary file 1 [file life-15-01940-s001.zip › life-3965317-supplementary.pdf]

**Supplementary material**

**Table S1**: Caves and bat species recorded

| No. | Cave ID | Species richness | Maximum abundance of bat species* | | | | | | | Total bat abundance |
|---|---|---|---|---|---|---|---|---|---|---|
| | | | *Coleura afra* | *Miniopterus* spp. | *Rhinolophus* spp. | *Rousettus aegyptiacus* | *Hipposideros caffer* | *Myonycteris angolensis* | *Nycteris macrotis* | |
| 1 | Mi | 3 | 0 | 0 | 4 | 0 | 0 | 55 | 5 | 64 |
| 2 | Kp | 3 | 0 | 0 | 342 | 0 | 225 | 0 | 276 | 843 |
| 3 | Kw | 5 | 0 | 684 | 645 | 528 | 120 | 0 | 134 | 2111 |
| 4 | Nw | 5 | 0 | 2060 | 680 | 2021 | 114 | 0 | 271 | 5146 |
| 5 | Wi | 2 | 0 | 3 | 135 | 0 | 0 | 0 | 0 | 138 |
| 6 | Ti | 4 | 0 | 0 | 20 | 236 | 32 | 24 | 0 | 312 |
| 7 | Ct | 3 | 0 | 0 | 38 | 0 | 26 | 0 | 23 | 87 |
| 8 | Tw | 2 | 0 | 37 | 0 | 0 | 0 | 0 | 6 | 43 |
| 9 | Kk | 2 | 0 | 0 | 0 | 0 | 11 | 32 | 0 | 43 |
| 10 | Ko | 1 | 0 | 0 | 47 | 0 | 0 | 0 | 0 | 47 |
| 11 | Lb | 2 | 0 | 0 | 57 | 0 | 0 | 0 | 66 | 123 |
| 12 | Kg | 2 | 0 | 0 | 108 | 0 | 0 | 0 | 58 | 166 |
| 13 | Kb | 1 | 0 | 0 | 0 | 0 | 0 | 0 | 141 | 141 |
| 14 | Mb | 2 | 1240 | 1620 | 0 | 0 | 0 | 0 | 0 | 2860 |

*Represents maximum number of different species of bats during the study period (2022-2024)

**Table S2**: Biotic vulnerability of the caves

| Cave geophysical features | | | | | | | | | |
|---|---|---|---|---|---|---|---|---|---|
| **Cave ID** | **Accessibility to cave sites** | **Cave openings** | **Effort of exploration** | **Tourism Activity** | **Cave Use** | **Land-use change activities within cave vicinity** | **Presence of temples and sacred structures** | **Biotic vulnerability Score** | **Biotic Vulnerability Index** |
| Mi | 1 | 1 | 1 | 1 | 1 | 1 | 1 | 1.00 | A |
| Kp | 1 | 1 | 2 | 2 | 1 | 1 | 1 | 1.29 | A |
| Kw | 1 | 1 | 1 | 3 | 1 | 1 | 1 | 1.29 | A |
| Nw | 1 | 1 | 1 | 3 | 1 | 1 | 1 | 1.29 | A |
| Wi | 1 | 1 | 1 | 3 | 1 | 1 | 1 | 1.29 | A |
| Ti | 1 | 1 | 1 | 3 | 1 | 1 | 1 | 1.29 | A |
| Ct | 3 | 3 | 4 | 4 | 4 | 1 | 4 | 3.29 | C |
| Tw | 1 | 1 | 1 | 3 | 1 | 1 | 1 | 1.29 | A |
| Kk | 1 | 1 | 1 | 3 | 1 | 1 | 1 | 1.29 | A |
| Ko | 1 | 1 | 1 | 3 | 1 | 1 | 1 | 1.29 | A |
| Lb | 2 | 2 | 1 | 3 | 1 | 1 | 2 | 1.71 | A |
| Kg | 1 | 3 | 3 | 3 | 2 | 1 | 2 | 2.14 | B |
| Kb | 1 | 3 | 3 | 3 | 2 | 1 | 2 | 2.14 | B |
| Mb | 1 | 3 | 3 | 3 | 2 | 1 | 2 | 2.14 | B |

**Table S3**: Landscape and human-induced activities that influence vulnerability of caves

| Geophysical and human activity features | Codes | Score | Scenario |
|---|---|---|---|
| Accessibility to cave sites | Acc | 1 | Easily accessible with no permit needed. The caves are very near to human settlements; easily accessible by a vehicle, motorcycle or easy walking distance. |
| | | 2 | No permit needed. Accessible with a motorcycle or two-wheeled vehicle. |
| | | 3 | Difficult to access, needs permission to enter, far from human settlements, with human trail. Requires trekking for under 8 h from the motorized vehicle accessible area. |
| | | 4 | Permit enter/explore is needed, no roads, no tracks, and trails, can be reached by trekking at least one day. |
| Cave openings | Co | 1 | Main openings are around 2 m tall and a meter wide. Two or more people can enter at the same time. |
| | | 2 | Anyone can enter, with more than 1 entrance but only one person at a time can pass through squat/crawl. |
| | | 3 | Difficult to enter, narrow openings but wide on the interior (needs to crawl and clamber). |
| | | 4 | Very difficult to pass, narrow entrance and narrow inside; needs special equipment to pass cave openings on vertical wall/vertical openings. |
| Effort of exploration | Eff | 1 | Easy to explore, no obstacles inside; can be explored by walking. |
| | | 2 | Easy to explore, with a minimal number of obstacles; crawling in some parts of the cave is needed. |
| | | 3 | Difficult to explore, many obstacles but no need for special skills. Squeezing and swimming may be needed |

| | | | |
|---|---|---|---|
| | | 4 | Very difficult to explore, many obstacles need special skills in exploration. May be dangerous to explore; special equipment like rappelling equipment/diving is necessary. |
| Tourism Activity | Tour | 1 | Tourism activity is very high. Frequent (at least 4× a month) visitation of large volume of visitors (more than 10 persons per group) per annum. |
| | | 2 | Intermittent (less than a 4× month) visitation of large volume (more than 10 persons) of tourists per year. |
| | | 3 | Occasional (less than 4× a month) visitation of small volume of the visitor (less than 10) per year |
| | | 4 | Not a potential tourist spot. No visitation at all. |
| Cave Use | CavUS | 1 | Intense cave use and exploitation. All of these disturbances are present in the cave: regular hunting of bats for bush meat and trade; high volumes of noise occur inside the cave; evidence of lighting or electric cables; mining of minerals; guano collection, settlement of domestic animals. |
| | | 2 | Minimal cave use is present. Two of the listed cave use mentioned above are present |
| | | 3 | Only single cave use and activity mentioned above is present in the cave. |
| | | 4 | All cave use mentioned above is absent. |
| Land-use change activities within cave vicinity | LandUs | 1 | All of these land use activities are present near the cave openings; multiple land-use activities are present nearby; monoculture plantations are present; forest conversion and mining/quarrying are also present. |
| | | 2 | Land-use is minimal; some land has been converted for small-scale agriculture; mining/quarrying is present. |
| | | 3 | Land-use mentioned above is present but too far; the forest is intercropped with small-scale agriculture; mining is absent. |
| | | 4 | Land-uses mentioned above are absent. Cave is located in a pristine forest. |
| Presence of temples and | Templ | 1 | Temples are present but highly used/visited for tourism. |

| | | | |
|---|---|---|---|
| sacred structures | | | |
| | | 2 | Temples are occasionally used for religious purposes. |
| | | 3 | Temples are present, high tourism, but entry to temples is prohibited |
| | | 4 | Temples are present but entry is completely prohibited |

**Table S4**: Conservation status of the bats

| Bat species name | Endemism | Score for endemism | Conservation status | Score for conservation status | Species site commonness index |
|---|---|---|---|---|---|
| *Coleura afra* | Regional Endemic (RE) (Africa - central, west and eastern) | 3 | Least Concern | 2 | 0.0714 |
| *Miniopterus* spp. | Regional Endemic (RE) (southeastern Africa) | 3 | Least Concern | 2 | 0.3571 |
| *Rhinolophus* spp. | Regional Endemic (RE) (Eastern Africa, tropical region, southern Africa, parts of northern Africa) | 3 | Least Concern | 2 | 0.7143 |
| *Rousettus aegyptiacus* | Widespread (NE) Africa and Asia | 2 | Least Concern | 2 | 0.2143 |
| *Hipposideros caffer* | Restricted to southeastern part of Africa | 5 | Least Concern | 2 | 0.4286 |
| *Myonycteris angolensis* | Restricted to tropical region | 5 | Least Concern | 2 | 0.2143 |
| *Nycteris macrotis* | Restricted to tropical region | 5 | Least Concern | 2 | 0.6429 |

**Table S5. Tests for differences in bat counts across species (within caves)**

| Approach | Test | Statistic | df | p-value | Interpretation |
|---|---|---|---|---|---|
| Rank-based (within-cave ranks) | Species effect | F = 3.01 | (6, 78) | 0.0106 | Counts differ across species within caves |
| Poisson FE (robust SE) | Joint species test | $\chi^2 = 44.67$ | 6 | <0.001 | Strong evidence of species differences |
| Negative binomial FE (robust SE) | Joint species test | $\chi^2 = 19.50$ | 6 | 0.0034 | Species differences persist under over-dispersion |

Bat counts varried significantly across species within caves (supported by both rank-based and count-regression approaches) (Table S5).

**Table S6. Tests for differences in bat counts across caves (overall)**

| Model | Test | Statistic | df | p-value | Interpretation |
|---|---|---|---|---|---|
| Poisson FE (robust SE) | Joint cave test | $\chi^2 = 179.33$ | 13 | <0.001 | Counts differ across caves overall |
| Negative binomial FE (robust SE) | Joint cave test | $\chi^2 = 63.23$ | 13 | <0.001 | Counts differ across caves overall |

**Model diagnostics (Poisson):** Both deviance and Pearson goodness-of-fit tests strongly rejected the Poisson fit ($p < 0.001$), consistent with over-dispersion; therefore, the negative binomial results are emphasised for inference. In conclusion, bat counts vary significantly across caves, even after controlling for species composition.

**Table S7. Species-specific variation across caves (Kruskal–Wallis tests)**

| Species | Kruskal–Wallis $\chi^2$ (df=13) with ties | p-value | Conclusion |
|---|---|---|---|
| Coleura afra | 13.00 | 0.4478 | No evidence of cave-to-cave variation detected |
| Miniopterus spp. | 13.00 | 0.4478 | No evidence detected |
| Rhinolophus spp. | 13.00 | 0.4478 | No evidence detected |
| Rousettus aegyptiacus | 13.00 | 0.4478 | No evidence detected |
| Hipposideros caffer | 13.00 | 0.4478 | No evidence detected |
| Myonycteris angolensis | 13.00 | 0.4478 | No evidence detected |
| Nycteris macrotis | 13.00 | 0.4478 | No evidence detected |

Each cave contributes only one observation per species (n=14 per species) and counts contain many ties (especially zeros). Kruskal–Wallis with ties therefore has limited power to detect differences across caves within individual species in this small dataset.

When analysed species-by-species using a non-parametric across-cave test, we did not detect statistically significant differences across caves for any single species. However, the pooled fixed-effects count models show strong overall cave differences after accounting for species, suggesting that cave-level differences are present in the aggregate but may be difficult to detect within any one species given sparsity and ties.
